# 4-Butyl­amino-3-nitro­benzoic acid

**DOI:** 10.1107/S1600536809014780

**Published:** 2009-04-25

**Authors:** Shivanagere Nagojappa Narendra Babu, Aisyah Saad Abdul Rahim, Hasnah Osman, Samuel Robinson Jebas, Hoong-Kun Fun

**Affiliations:** a School of Pharmaceutical Sciences, Universiti Sains Malaysia, 11800 USM, Penang, Malaysia; b School of Chemical Sciences, Universiti Sains Malaysia, 11800 USM, Penang, Malaysia; c X-ray Crystallography Unit, School of Physics, Universiti Sains Malaysia, 11800 USM, Penang, Malaysia

## Abstract

The asymmetric unit of the title compound, C_11_H_14_N_2_O_4_, comprises four crystallographically independent mol­ecules (*A*, *B*, *C* and *D*) with similar geometries. In each mol­ecule, the butyl­amino side chain is in an extended conformation, and the carboxyl and butyl­amino groups are almost coplanar with the attached benzene ring; the nitro group is slightly twisted away from the benzene ring. In the asymmetric unit, the benzene rings of mol­ecules *A*, *B* and *C* are stacked parallel to one another, with a centroid–centroid distance of 3.6197 (11) or 3.6569 (11) Å, indicating π–π inter­actions. An intra­molecular N—H⋯O hydrogen bond is observed in each independent mol­ecule. In addition to the π–π inter­actions, the crystal packing is consolidated by inter­molecular O—H⋯O and C—H⋯O hydrogen bonds and C—H⋯π inter­actions. The crystal studied was a non-merohedral twin. The minor twin component refined to a value of 0.290 (1).

## Related literature

For the synthesis of nitro­benzoic acid derivatives, see: Ishida *et al.* (2006 ▶); Mohd Maidin *et al.* (2008 ▶). For bond-length data, see: Allen *et al.* (1987 ▶). For the stability of the temperature controller used for the data collection, see: Cosier & Glazer (1986 ▶).
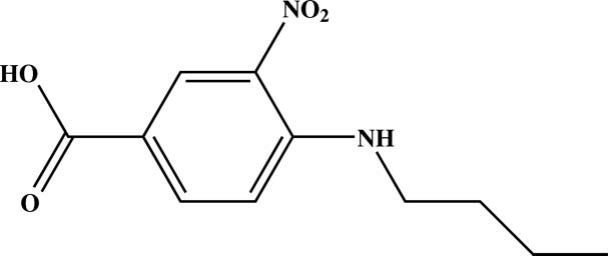


## Experimental

### 

#### Crystal data

C_11_H_14_N_2_O_4_
*M* *_r_* = 238.24
Monoclinic, 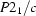

*a* = 14.5188 (6) Å
*b* = 13.8801 (6) Å
*c* = 22.5694 (9) Å
β = 90.233 (2)°
*V* = 4548.2 (3) Å^3^
*Z* = 16
Mo *K*α radiation
μ = 0.11 mm^−1^
*T* = 100 K
0.52 × 0.19 × 0.13 mm

#### Data collection

Bruker SMART APEXII CCD area-detector diffractometer
Absorption correction: multi-scan (*SADABS*; Bruker, 2005 ▶) *T*_min_ = 0.946, *T*_max_ = 0.986
137935 measured reflections
15056 independent reflections
11743 reflections with *I* > 2σ(*I*)
*R*_int_ = 0.066

#### Refinement

*R*[*F* ^2^ > 2σ(*F* ^2^)] = 0.064
*wR*(*F* ^2^) = 0.163
*S* = 1.06
15056 reflections
622 parameters
H-atom parameters constrained
Δρ_max_ = 0.41 e Å^−3^
Δρ_min_ = −0.23 e Å^−3^

### 

Data collection: *APEX2* (Bruker, 2005 ▶); cell refinement: *SAINT* (Bruker, 2005 ▶); data reduction: *SAINT*; program(s) used to solve structure: *SHELXTL* (Sheldrick, 2008 ▶); program(s) used to refine structure: *SHELXTL*; molecular graphics: *SHELXTL*; software used to prepare material for publication: *SHELXTL* and *PLATON* (Spek, 2009 ▶).

## Supplementary Material

Crystal structure: contains datablocks global, I. DOI: 10.1107/S1600536809014780/ci2784sup1.cif

Structure factors: contains datablocks I. DOI: 10.1107/S1600536809014780/ci2784Isup2.hkl

Additional supplementary materials:  crystallographic information; 3D view; checkCIF report

## Figures and Tables

**Table 1:** Hydrogen-bond geometry (Å, °)

*D*—H⋯*A*	*D*—H	H⋯*A*	*D*⋯*A*	*D*—H⋯*A*
O2*A*—H2*A*⋯O1*A*^i^	0.82	1.80	2.613 (2)	168
O2*B*—H2*B*⋯O1*D*^ii^	0.82	1.81	2.624 (2)	172
O2*C*—H2*C*⋯O1*C*^iii^	0.82	1.80	2.619 (2)	175
O2*D*—H2*D*⋯O1*B*^iv^	0.82	1.80	2.612 (2)	173
C1*A*—H1*AA*⋯O3*A*^v^	0.93	2.46	3.290 (2)	149
C1*C*—H1*CA*⋯O3*C*^vi^	0.93	2.44	3.256 (2)	146
C1*D*—H1*DA*⋯O3*B*^vii^	0.93	2.40	3.227 (2)	148
N2*A*—H2*AB*⋯O4*A*	0.86	2.02	2.656 (2)	130
N2*B*—H2*BB*⋯O4*B*	0.86	2.01	2.651 (2)	130
N2*C*—H2*CB*⋯O4*C*	0.86	2.02	2.649 (2)	129
N2*D*—H2*DB*⋯O4*D*	0.86	2.01	2.649 (2)	130
C1*B*—H1*BA*⋯O3*D*	0.93	2.49	3.311 (2)	147
C7*D*—H7*DA*⋯*Cg*1^v^	0.97	2.81	3.584 (2)	137
C7*C*—H7*CB*⋯*Cg*2^viii^	0.97	2.88	3.621 (2)	134

Symmetry codes: (i) 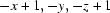; (ii) 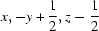; (iii) 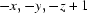; (iv) 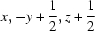; (v) 
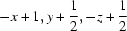
; (vi) 
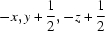
; (vii) 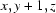; (viii) 
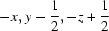
. *Cg*1 and *Cg*2 are the centroids of the C1*A*–C6*A* and C1*D*–C6*D* rings, respectively.

**Fig. 1.**
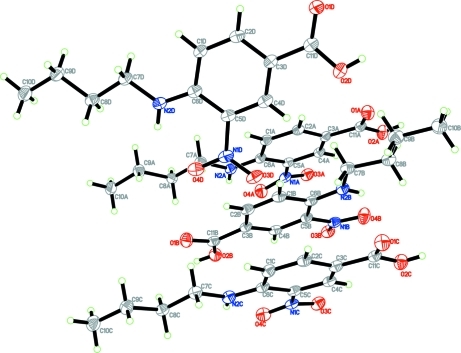
The asymmetric unit of the title compound, showing 50% probability displacement ellipsoids and the atom-numbering scheme.

**Fig. 2.**
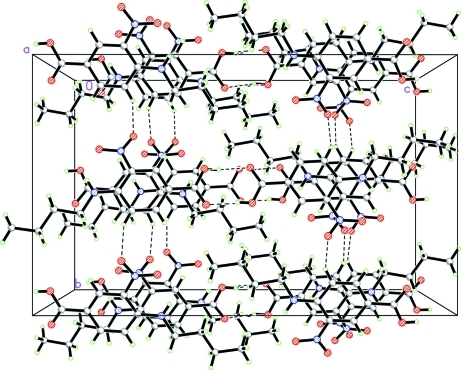
The crystal packing of the title compound, viewed along the a axis. Dashed lines indicate the hydrogen bonding.

## supplementary crystallographic information

### Comment

Synthesis of biologically active heterocyclic scaffolds can be accessed conveniently *via* nitro benzoic acid intermediates (Ishida *et al.*, 2006). We synthesized the title compound as an intermediate, and herein we report its crystal structure.

The asymmetric unit of of the title compound (Fig.1) comprises of four crystallographically independent molecules (A, B, C & D) with similar geometries. The bond lengths (Allen *et al.*, 1987) and angles have normal values. In each of these molecules, the butylamino side chain is in an extended conformation. The carboxyl and butylamino groups are almost coplanar with the attached bezene ring. The nitro group is slightly twisted away from the benzene ring, with the dihedral angle between them being 13.79 (10)° in molecule A [10.39 (10)° in B, 5.88 (10)° in C and 9.52 (10)° in D]. In the asymmetric unit, molecules A, B and C are stacked almost parallel to one another but the orientation of the molecule D is different. The benzene ring of molecule A forms dihedral angles of 2.93 (9) and 1.95 (9)°, respectively, with benzene rings of molecules B and C. The benzene ring of molecule D forms dihedral angles of 49.37 (9), 47.22 (9) and 47.73 (9)°, respectively, with benzene rings of molecules A, B and C.

An intramolecular N—H···O hydrogen bond is observed in each independent molecule. In the asymmetric unit, molecules B and D are linked via a C—H···O hydrogen bond. The crystal packing (Fig. 2) is consolidated by intermolecular O—H···O and C—H···O hydrogen bonds and intermolecular C—H···π interactions (Table 1). In addition, π-π interactions are observed between the benzene rings of molecules A, B and C, with Cg1···Cg2 and Cg2···Cg3 distances of 3.6197 (11) Å and 3.6569 (11) Å, respectively; Cg1, Cg2 and Cg3 are centroids of the C1A-C6A, C1B-C6B and C1C-C6B benzene rings, respectively.

### Experimental

A mixture of ethyl 4-butylamino-3-nitro-benzoate (0.5 g, 0.0018 mol) (Mohd Maidin *et al.*, 2008) and KOH (0.10 g, 0.0018 mol) was refluxed in aqueous ethanol (10 ml) for 3 h. After completion of the reaction, ethanol was distilled off and the reaction mixture was diluted with water (15 ml). The aqueous layer was washed with dichloromethane (5 × 2 ml) and acidified with concentrated hydrochloric acid to afford yellow precipitate as the crude product. Recrystallization of the crude product with hot ethyl acetate gave the title compound as yellow needles.

### Refinement

H atoms were positioned geometrically [C-H = 0.96–0.97 Å; O-H = 0.82 Å and N-H = 0.86 Å] and refined using a riding model, with *U*_iso_(H) = 1.2*U*_eq_(C,N) and 1.5*U*_eq_(O,C_methyl_). A rotating–group model was used for the methyl groups. The crystal studied was a non-merohedral twin. The minor twin component refined to a value of 0.290 (1).

### Crystal data

**Table:**

C_11_H_14_N_2_O_4_	*F*(000) = 2016
*M**_r_* = 238.24	*D*_x_ = 1.392 Mg m^−^^3^
Monoclinic, *P*2_1_/*c*	Mo *K*α radiation, λ = 0.71073 Å
Hall symbol: -P 2ybc	Cell parameters from 9852 reflections
*a* = 14.5188 (6) Å	θ = 2.2–31.6°
*b* = 13.8801 (6) Å	µ = 0.11 mm^−^^1^
*c* = 22.5694 (9) Å	*T* = 100 K
β = 90.233 (2)°	Needle, yellow
*V* = 4548.2 (3) Å^3^	0.52 × 0.19 × 0.13 mm
*Z* = 16	

### Data collection

**Table:**

Bruker SMART APEXII CCD area-detector diffractometer	15056 independent reflections
Radiation source: fine-focus sealed tube	11743 reflections with *I* > 2σ(*I*)
graphite	*R*_int_ = 0.066
φ and ω scans	θ_max_ = 31.5°, θ_min_ = 0.9°
Absorption correction: multi-scan (*SADABS*; Bruker, 2005)	*h* = −21→20
*T*_min_ = 0.946, *T*_max_ = 0.986	*k* = −20→20
137935 measured reflections	*l* = −33→33

### Refinement

**Table:**

Refinement on *F*^2^	Primary atom site location: structure-invariant direct methods
Least-squares matrix: full	Secondary atom site location: difference Fourier map
*R*[*F*^2^ > 2σ(*F*^2^)] = 0.064	Hydrogen site location: inferred from neighbouring sites
*wR*(*F*^2^) = 0.163	H-atom parameters constrained
*S* = 1.06	*w* = 1/[σ^2^(*F*_o_^2^) + (0.0774*P*)^2^ + 1.2697*P*] where *P* = (*F*_o_^2^ + 2*F*_c_^2^)/3
15056 reflections	(Δ/σ)_max_ = 0.001
622 parameters	Δρ_max_ = 0.41 e Å^−^^3^
0 restraints	Δρ_min_ = −0.23 e Å^−^^3^

### Special details

**Table:**

Experimental. The crystal was placed in the cold stream of an Oxford Cyrosystems Cobra open-flow nitrogen cryostat (Cosier & Glazer, 1986) operating at 100.0 (1) K.
Geometry. All e.s.d.'s (except the e.s.d. in the dihedral angle between two l.s. planes) are estimated using the full covariance matrix. The cell e.s.d.'s are taken into account individually in the estimation of e.s.d.'s in distances, angles and torsion angles; correlations between e.s.d.'s in cell parameters are only used when they are defined by crystal symmetry. An approximate (isotropic) treatment of cell e.s.d.'s is used for estimating e.s.d.'s involving l.s. planes.
Refinement. Refinement of *F*^2^ against ALL reflections. The weighted *R*-factor *wR* and goodness of fit *S* are based on *F*^2^, conventional *R*-factors *R* are based on *F*, with *F* set to zero for negative *F*^2^. The threshold expression of *F*^2^ > σ(*F*^2^) is used only for calculating *R*-factors(gt) *etc*. and is not relevant to the choice of reflections for refinement. *R*-factors based on *F*^2^ are statistically about twice as large as those based on *F*, and *R*- factors based on ALL data will be even larger.

### Fractional atomic coordinates and isotropic or equivalent isotropic displacement parameters (Å^2^)

**Table:**

	*x*	*y*	*z*	*U*_iso_*/*U*_eq_	
O1A	0.47049 (11)	0.07879 (11)	0.44554 (7)	0.0282 (3)	
O2A	0.54315 (11)	−0.06355 (10)	0.43941 (7)	0.0279 (3)	
H2A	0.5392	−0.0601	0.4756	0.042*	
O3A	0.63546 (10)	−0.18919 (10)	0.25553 (7)	0.0274 (3)	
O4A	0.57916 (12)	−0.13846 (11)	0.17198 (7)	0.0311 (3)	
N1A	0.59022 (11)	−0.13029 (11)	0.22614 (8)	0.0224 (3)	
N2A	0.50963 (11)	0.03823 (11)	0.16551 (8)	0.0209 (3)	
H2AB	0.5247	−0.0119	0.1453	0.025*	
C1A	0.48194 (13)	0.10971 (13)	0.26041 (9)	0.0205 (4)	
H1AA	0.4617	0.1660	0.2421	0.025*	
C2A	0.48021 (13)	0.10388 (13)	0.32115 (9)	0.0200 (4)	
H2AA	0.4572	0.1552	0.3430	0.024*	
C3A	0.51283 (13)	0.02098 (13)	0.35082 (9)	0.0195 (4)	
C4A	0.54881 (12)	−0.05358 (13)	0.31793 (9)	0.0200 (4)	
H4AA	0.5726	−0.1076	0.3370	0.024*	
C5A	0.54979 (13)	−0.04862 (13)	0.25652 (9)	0.0194 (4)	
C6A	0.51375 (12)	0.03214 (13)	0.22463 (9)	0.0192 (4)	
C7A	0.48124 (14)	0.12411 (13)	0.13296 (9)	0.0213 (4)	
H7AA	0.5219	0.1772	0.1427	0.026*	
H7AB	0.4192	0.1419	0.1443	0.026*	
C8A	0.48435 (14)	0.10498 (14)	0.06642 (9)	0.0216 (4)	
H8AA	0.5461	0.0854	0.0555	0.026*	
H8AB	0.4428	0.0524	0.0569	0.026*	
C9A	0.45733 (17)	0.19300 (14)	0.03070 (10)	0.0282 (4)	
H9AA	0.5001	0.2449	0.0393	0.034*	
H9AB	0.3964	0.2139	0.0427	0.034*	
C10A	0.45701 (18)	0.17360 (16)	−0.03620 (10)	0.0314 (5)	
H10A	0.4393	0.2311	−0.0569	0.047*	
H10B	0.4140	0.1230	−0.0451	0.047*	
H10C	0.5176	0.1545	−0.0485	0.047*	
C11A	0.50779 (13)	0.01307 (13)	0.41582 (9)	0.0208 (4)	
O1B	0.23328 (11)	0.07949 (10)	0.09745 (7)	0.0287 (3)	
O2B	0.29894 (11)	−0.06670 (10)	0.10319 (7)	0.0273 (3)	
H2B	0.2955	−0.0626	0.0670	0.041*	
O3B	0.37906 (10)	−0.20336 (9)	0.28766 (7)	0.0255 (3)	
O4B	0.33005 (12)	−0.14787 (11)	0.37108 (7)	0.0330 (4)	
N1B	0.33887 (11)	−0.14104 (11)	0.31669 (8)	0.0219 (3)	
N2B	0.26497 (11)	0.03023 (11)	0.37760 (7)	0.0218 (3)	
H2BB	0.2806	−0.0196	0.3979	0.026*	
C1B	0.23970 (13)	0.10337 (13)	0.28236 (9)	0.0223 (4)	
H1BA	0.2196	0.1594	0.3008	0.027*	
C2B	0.24000 (13)	0.09963 (13)	0.22208 (9)	0.0216 (4)	
H2BA	0.2198	0.1527	0.2004	0.026*	
C3B	0.27041 (13)	0.01657 (13)	0.19202 (9)	0.0201 (4)	
C4B	0.30298 (13)	−0.06058 (13)	0.22507 (9)	0.0202 (4)	
H4BA	0.3251	−0.1150	0.2058	0.024*	
C5B	0.30286 (13)	−0.05742 (13)	0.28641 (9)	0.0201 (4)	
C6B	0.26909 (12)	0.02464 (13)	0.31820 (9)	0.0197 (4)	
C7B	0.23541 (14)	0.11643 (13)	0.40947 (9)	0.0231 (4)	
H7BA	0.1747	0.1354	0.3957	0.028*	
H7BB	0.2777	0.1689	0.4013	0.028*	
C8B	0.23267 (14)	0.09792 (13)	0.47553 (9)	0.0227 (4)	
H8BA	0.2938	0.0809	0.4894	0.027*	
H8BB	0.1920	0.0441	0.4835	0.027*	
C9B	0.19895 (16)	0.18646 (14)	0.50902 (10)	0.0280 (4)	
H9BA	0.1372	0.2024	0.4956	0.034*	
H9BB	0.2387	0.2406	0.4999	0.034*	
C10B	0.19780 (17)	0.17118 (15)	0.57604 (10)	0.0306 (5)	
H10D	0.1767	0.2289	0.5951	0.046*	
H10E	0.2589	0.1562	0.5897	0.046*	
H10F	0.1571	0.1189	0.5855	0.046*	
C11B	0.26655 (13)	0.01152 (13)	0.12696 (9)	0.0212 (4)	
O1C	−0.01824 (11)	0.07275 (10)	0.44434 (7)	0.0273 (3)	
O2C	0.04469 (11)	−0.07447 (10)	0.44094 (7)	0.0272 (3)	
H2C	0.0378	−0.0709	0.4769	0.041*	
O3C	0.13147 (10)	−0.21295 (9)	0.25882 (7)	0.0262 (3)	
O4C	0.09802 (10)	−0.15093 (10)	0.17316 (7)	0.0273 (3)	
N1C	0.09807 (11)	−0.14756 (11)	0.22802 (8)	0.0209 (3)	
N2C	0.02634 (11)	0.02410 (11)	0.16522 (8)	0.0217 (3)	
H2CB	0.0461	−0.0239	0.1448	0.026*	
C1C	−0.00491 (13)	0.09561 (13)	0.25964 (9)	0.0213 (4)	
H1CA	−0.0244	0.1515	0.2407	0.026*	
C2C	−0.00795 (13)	0.09166 (13)	0.32017 (10)	0.0217 (4)	
H2CA	−0.0297	0.1444	0.3413	0.026*	
C3C	0.02148 (13)	0.00852 (13)	0.35102 (9)	0.0204 (4)	
C4C	0.05534 (13)	−0.06864 (13)	0.31884 (10)	0.0208 (4)	
H4CA	0.0757	−0.1236	0.3385	0.025*	
C5C	0.05902 (12)	−0.06455 (12)	0.25737 (9)	0.0196 (4)	
C6C	0.02695 (12)	0.01732 (13)	0.22475 (9)	0.0199 (4)	
C7C	−0.00657 (14)	0.11007 (14)	0.13378 (9)	0.0233 (4)	
H7CA	0.0336	0.1640	0.1428	0.028*	
H7CB	−0.0680	0.1262	0.1474	0.028*	
C8C	−0.00883 (14)	0.09388 (14)	0.06709 (9)	0.0218 (4)	
H8CA	−0.0462	0.0378	0.0582	0.026*	
H8CB	0.0531	0.0816	0.0530	0.026*	
C9C	−0.04841 (15)	0.18165 (14)	0.03515 (10)	0.0262 (4)	
H9CA	−0.1104	0.1932	0.0493	0.031*	
H9CB	−0.0115	0.2377	0.0450	0.031*	
C10C	−0.05119 (18)	0.17027 (16)	−0.03199 (10)	0.0319 (5)	
H10G	−0.0751	0.2281	−0.0496	0.048*	
H10H	−0.0901	0.1169	−0.0423	0.048*	
H10I	0.0099	0.1587	−0.0464	0.048*	
C11C	0.01442 (13)	0.00514 (13)	0.41585 (9)	0.0209 (4)	
O1D	0.28378 (10)	0.57136 (10)	0.48734 (7)	0.0265 (3)	
O2D	0.21231 (11)	0.42785 (10)	0.48256 (7)	0.0274 (3)	
H2D	0.2208	0.4301	0.5185	0.041*	
O3D	0.11204 (11)	0.29907 (10)	0.29922 (7)	0.0289 (3)	
O4D	0.15340 (10)	0.35615 (10)	0.21422 (7)	0.0284 (3)	
N1D	0.15116 (11)	0.36082 (12)	0.26892 (8)	0.0224 (3)	
N2D	0.22872 (11)	0.52963 (11)	0.20761 (7)	0.0205 (3)	
H2DB	0.2094	0.4813	0.1873	0.025*	
C1D	0.26478 (13)	0.59870 (13)	0.30210 (9)	0.0203 (4)	
H1DA	0.2857	0.6541	0.2833	0.024*	
C2D	0.26970 (13)	0.59351 (13)	0.36282 (9)	0.0214 (4)	
H2DA	0.2938	0.6450	0.3842	0.026*	
C3D	0.23836 (13)	0.51058 (13)	0.39304 (9)	0.0197 (4)	
C4D	0.20020 (13)	0.43621 (13)	0.36041 (9)	0.0223 (4)	
H4DA	0.1780	0.3820	0.3799	0.027*	
C5D	0.19448 (13)	0.44113 (13)	0.29900 (9)	0.0199 (4)	
C6D	0.22884 (12)	0.52248 (13)	0.26689 (9)	0.0205 (4)	
C7D	0.25981 (13)	0.61532 (13)	0.17588 (9)	0.0214 (4)	
H7DA	0.3226	0.6302	0.1875	0.026*	
H7DB	0.2213	0.6697	0.1865	0.026*	
C8D	0.25540 (14)	0.59971 (13)	0.10953 (9)	0.0224 (4)	
H8DA	0.1922	0.5874	0.0978	0.027*	
H8DB	0.2918	0.5436	0.0992	0.027*	
C9D	0.29151 (16)	0.68734 (14)	0.07614 (10)	0.0278 (4)	
H9DA	0.2536	0.7427	0.0856	0.033*	
H9DB	0.3538	0.7011	0.0894	0.033*	
C10D	0.29172 (18)	0.67307 (15)	0.00921 (10)	0.0317 (5)	
H10J	0.3138	0.7306	−0.0096	0.048*	
H10K	0.2303	0.6596	−0.0042	0.048*	
H10L	0.3313	0.6201	−0.0007	0.048*	
C11D	0.24632 (13)	0.50516 (13)	0.45793 (9)	0.0213 (4)	

### Atomic displacement parameters (Å^2^)

**Table:**

	*U*^11^	*U*^22^	*U*^33^	*U*^12^	*U*^13^	*U*^23^
O1A	0.0316 (8)	0.0284 (7)	0.0246 (8)	0.0040 (6)	0.0033 (6)	−0.0014 (6)
O2A	0.0329 (8)	0.0260 (7)	0.0249 (8)	0.0040 (6)	0.0017 (6)	0.0066 (6)
O3A	0.0271 (7)	0.0179 (6)	0.0371 (9)	0.0041 (5)	0.0005 (6)	0.0016 (6)
O4A	0.0424 (9)	0.0253 (7)	0.0256 (8)	0.0061 (6)	0.0002 (7)	−0.0037 (6)
N1A	0.0208 (8)	0.0168 (7)	0.0295 (9)	−0.0010 (6)	0.0027 (7)	0.0001 (6)
N2A	0.0207 (8)	0.0189 (7)	0.0229 (9)	−0.0003 (6)	0.0003 (6)	0.0000 (6)
C1A	0.0182 (8)	0.0164 (8)	0.0270 (11)	0.0000 (6)	−0.0016 (7)	0.0022 (7)
C2A	0.0163 (8)	0.0186 (8)	0.0251 (10)	−0.0014 (6)	0.0019 (7)	−0.0014 (7)
C3A	0.0170 (8)	0.0190 (8)	0.0224 (10)	−0.0030 (6)	0.0019 (7)	0.0028 (7)
C4A	0.0163 (8)	0.0174 (8)	0.0264 (10)	−0.0016 (6)	0.0004 (7)	0.0018 (7)
C5A	0.0160 (8)	0.0163 (8)	0.0260 (10)	−0.0013 (6)	0.0015 (7)	−0.0003 (7)
C6A	0.0149 (8)	0.0191 (8)	0.0237 (10)	−0.0019 (6)	0.0020 (7)	0.0015 (7)
C7A	0.0213 (9)	0.0183 (8)	0.0245 (10)	−0.0002 (7)	−0.0010 (7)	−0.0008 (7)
C8A	0.0226 (9)	0.0206 (8)	0.0216 (10)	−0.0002 (7)	−0.0015 (7)	0.0000 (7)
C9A	0.0375 (12)	0.0217 (9)	0.0253 (11)	0.0030 (8)	−0.0012 (9)	0.0002 (8)
C10A	0.0430 (13)	0.0249 (10)	0.0261 (11)	−0.0019 (9)	−0.0025 (9)	0.0039 (8)
C11A	0.0182 (8)	0.0201 (8)	0.0241 (10)	−0.0033 (7)	0.0006 (7)	0.0015 (7)
O1B	0.0297 (8)	0.0265 (7)	0.0298 (8)	0.0041 (6)	−0.0016 (6)	0.0041 (6)
O2B	0.0349 (8)	0.0223 (7)	0.0248 (8)	0.0011 (6)	0.0011 (7)	−0.0024 (6)
O3B	0.0264 (7)	0.0165 (6)	0.0335 (8)	0.0033 (5)	0.0015 (6)	−0.0009 (6)
O4B	0.0440 (9)	0.0250 (7)	0.0302 (8)	0.0064 (6)	0.0038 (7)	0.0065 (6)
N1B	0.0206 (8)	0.0149 (7)	0.0303 (9)	−0.0009 (6)	−0.0013 (7)	0.0001 (6)
N2B	0.0224 (8)	0.0169 (7)	0.0261 (8)	0.0019 (6)	0.0006 (7)	−0.0002 (6)
C1B	0.0192 (9)	0.0150 (8)	0.0328 (11)	0.0015 (6)	0.0001 (8)	−0.0042 (7)
C2B	0.0157 (8)	0.0186 (8)	0.0304 (11)	0.0003 (6)	−0.0005 (7)	0.0010 (7)
C3B	0.0148 (8)	0.0188 (8)	0.0267 (10)	−0.0023 (6)	0.0018 (7)	0.0008 (7)
C4B	0.0147 (8)	0.0158 (8)	0.0300 (11)	−0.0012 (6)	0.0006 (7)	−0.0019 (7)
C5B	0.0172 (8)	0.0157 (7)	0.0276 (10)	−0.0001 (6)	−0.0010 (7)	0.0014 (7)
C6B	0.0138 (8)	0.0179 (8)	0.0275 (10)	−0.0025 (6)	0.0012 (7)	0.0000 (7)
C7B	0.0211 (9)	0.0195 (8)	0.0287 (10)	0.0005 (7)	0.0002 (8)	−0.0027 (8)
C8B	0.0202 (9)	0.0191 (8)	0.0287 (10)	0.0015 (7)	0.0012 (8)	−0.0025 (8)
C9B	0.0309 (11)	0.0218 (9)	0.0312 (11)	0.0011 (8)	0.0012 (9)	−0.0025 (8)
C10B	0.0390 (12)	0.0239 (9)	0.0290 (11)	−0.0003 (9)	0.0026 (9)	−0.0037 (8)
C11B	0.0157 (8)	0.0193 (8)	0.0285 (10)	−0.0017 (7)	0.0007 (7)	−0.0002 (7)
O1C	0.0317 (8)	0.0221 (7)	0.0282 (8)	0.0061 (6)	0.0018 (6)	−0.0016 (6)
O2C	0.0331 (8)	0.0204 (7)	0.0281 (8)	0.0032 (6)	0.0015 (7)	0.0023 (6)
O3C	0.0267 (7)	0.0160 (6)	0.0358 (8)	0.0032 (5)	0.0022 (6)	0.0019 (6)
O4C	0.0281 (8)	0.0236 (7)	0.0303 (8)	0.0024 (6)	0.0025 (6)	−0.0055 (6)
N1C	0.0167 (7)	0.0154 (7)	0.0306 (9)	−0.0019 (5)	0.0015 (6)	−0.0012 (6)
N2C	0.0218 (8)	0.0166 (7)	0.0268 (9)	0.0027 (6)	0.0019 (7)	−0.0012 (6)
C1C	0.0188 (8)	0.0175 (8)	0.0276 (11)	0.0014 (6)	0.0015 (7)	0.0022 (7)
C2C	0.0181 (8)	0.0160 (8)	0.0309 (11)	0.0002 (6)	0.0004 (8)	−0.0018 (7)
C3C	0.0166 (8)	0.0170 (8)	0.0277 (10)	−0.0006 (6)	0.0011 (7)	0.0003 (7)
C4C	0.0170 (8)	0.0161 (8)	0.0295 (11)	−0.0023 (6)	−0.0005 (7)	0.0008 (7)
C5C	0.0162 (8)	0.0142 (7)	0.0283 (10)	−0.0011 (6)	0.0022 (7)	−0.0011 (7)
C6C	0.0137 (8)	0.0176 (8)	0.0283 (11)	−0.0021 (6)	0.0019 (7)	−0.0001 (7)
C7C	0.0204 (9)	0.0200 (8)	0.0295 (11)	0.0018 (7)	0.0001 (8)	0.0000 (8)
C8C	0.0201 (9)	0.0199 (8)	0.0254 (10)	0.0000 (7)	0.0015 (7)	−0.0003 (7)
C9C	0.0288 (10)	0.0201 (9)	0.0298 (11)	0.0029 (7)	0.0012 (8)	0.0013 (8)
C10C	0.0395 (12)	0.0248 (10)	0.0315 (12)	0.0023 (9)	−0.0011 (10)	0.0027 (9)
C11C	0.0168 (8)	0.0188 (8)	0.0270 (10)	−0.0022 (7)	0.0003 (7)	0.0012 (7)
O1D	0.0279 (8)	0.0255 (7)	0.0261 (8)	−0.0049 (6)	0.0008 (6)	0.0000 (6)
O2D	0.0314 (8)	0.0233 (7)	0.0274 (8)	−0.0038 (6)	0.0018 (7)	0.0057 (6)
O3D	0.0279 (7)	0.0193 (6)	0.0396 (9)	−0.0054 (5)	0.0020 (7)	0.0019 (6)
O4D	0.0275 (8)	0.0247 (7)	0.0330 (9)	−0.0030 (6)	−0.0003 (6)	−0.0042 (6)
N1D	0.0168 (7)	0.0169 (7)	0.0333 (10)	0.0006 (6)	0.0005 (7)	−0.0003 (6)
N2D	0.0196 (7)	0.0174 (7)	0.0245 (8)	−0.0010 (6)	0.0017 (6)	−0.0015 (6)
C1D	0.0184 (8)	0.0164 (8)	0.0261 (10)	−0.0003 (6)	0.0020 (7)	0.0017 (7)
C2D	0.0174 (8)	0.0174 (8)	0.0295 (10)	0.0006 (6)	0.0019 (7)	−0.0024 (7)
C3D	0.0168 (8)	0.0177 (8)	0.0245 (9)	0.0014 (6)	0.0037 (7)	0.0007 (7)
C4D	0.0159 (8)	0.0174 (8)	0.0336 (11)	0.0014 (6)	0.0018 (8)	0.0019 (7)
C5D	0.0152 (8)	0.0162 (8)	0.0283 (10)	0.0006 (6)	0.0023 (7)	−0.0021 (7)
C6D	0.0140 (8)	0.0195 (8)	0.0280 (10)	0.0018 (6)	0.0025 (7)	−0.0007 (7)
C7D	0.0191 (9)	0.0192 (8)	0.0260 (10)	0.0000 (7)	0.0029 (7)	0.0002 (7)
C8D	0.0198 (9)	0.0186 (8)	0.0287 (10)	0.0002 (7)	0.0017 (8)	−0.0005 (7)
C9D	0.0324 (11)	0.0212 (9)	0.0299 (11)	−0.0019 (8)	0.0019 (9)	0.0021 (8)
C10D	0.0410 (13)	0.0240 (9)	0.0303 (12)	0.0002 (9)	0.0007 (10)	0.0029 (8)
C11D	0.0166 (8)	0.0176 (8)	0.0297 (10)	0.0038 (6)	0.0020 (7)	0.0015 (7)

### Geometric parameters (Å, °)

**Table:**

O1A—C11A	1.256 (2)	O1C—C11C	1.234 (2)
O2A—C11A	1.295 (2)	O2C—C11C	1.316 (2)
O2A—H2A	0.82	O2C—H2C	0.82
O3A—N1A	1.239 (2)	O3C—N1C	1.241 (2)
O4A—N1A	1.238 (2)	O4C—N1C	1.239 (2)
N1A—C5A	1.450 (2)	N1C—C5C	1.446 (2)
N2A—C6A	1.338 (3)	N2C—C6C	1.347 (3)
N2A—C7A	1.459 (2)	N2C—C7C	1.467 (2)
N2A—H2AB	0.86	N2C—H2CB	0.86
C1A—C2A	1.374 (3)	C1C—C2C	1.368 (3)
C1A—C6A	1.424 (3)	C1C—C6C	1.420 (3)
C1A—H1AA	0.93	C1C—H1CA	0.93
C2A—C3A	1.412 (3)	C2C—C3C	1.413 (3)
C2A—H2AA	0.93	C2C—H2CA	0.93
C3A—C4A	1.378 (3)	C3C—C4C	1.385 (3)
C3A—C11A	1.473 (3)	C3C—C11C	1.468 (3)
C4A—C5A	1.388 (3)	C4C—C5C	1.390 (3)
C4A—H4AA	0.93	C4C—H4CA	0.93
C5A—C6A	1.430 (3)	C5C—C6C	1.431 (3)
C7A—C8A	1.526 (3)	C7C—C8C	1.522 (3)
C7A—H7AA	0.97	C7C—H7CA	0.97
C7A—H7AB	0.97	C7C—H7CB	0.97
C8A—C9A	1.515 (3)	C8C—C9C	1.527 (3)
C8A—H8AA	0.97	C8C—H8CA	0.97
C8A—H8AB	0.97	C8C—H8CB	0.97
C9A—C10A	1.534 (3)	C9C—C10C	1.524 (3)
C9A—H9AA	0.97	C9C—H9CA	0.97
C9A—H9AB	0.97	C9C—H9CB	0.97
C10A—H10A	0.96	C10C—H10G	0.96
C10A—H10B	0.96	C10C—H10H	0.96
C10A—H10C	0.96	C10C—H10I	0.96
O1B—C11B	1.251 (2)	O1D—C11D	1.256 (2)
O2B—C11B	1.300 (2)	O2D—C11D	1.306 (2)
O2B—H2B	0.82	O2D—H2D	0.82
O3B—N1B	1.233 (2)	O3D—N1D	1.236 (2)
O4B—N1B	1.238 (2)	O4D—N1D	1.237 (2)
N1B—C5B	1.444 (2)	N1D—C5D	1.447 (2)
N2B—C6B	1.344 (3)	N2D—C6D	1.342 (3)
N2B—C7B	1.461 (2)	N2D—C7D	1.461 (2)
N2B—H2BB	0.86	N2D—H2DB	0.86
C1B—C2B	1.362 (3)	C1D—C2D	1.374 (3)
C1B—C6B	1.424 (3)	C1D—C6D	1.421 (3)
C1B—H1BA	0.93	C1D—H1DA	0.93
C2B—C3B	1.410 (3)	C2D—C3D	1.414 (3)
C2B—H2BA	0.93	C2D—H2DA	0.93
C3B—C4B	1.387 (3)	C3D—C4D	1.383 (3)
C3B—C11B	1.471 (3)	C3D—C11D	1.470 (3)
C4B—C5B	1.385 (3)	C4D—C5D	1.390 (3)
C4B—H4BA	0.93	C4D—H4DA	0.93
C5B—C6B	1.434 (3)	C5D—C6D	1.432 (3)
C7B—C8B	1.514 (3)	C7D—C8D	1.514 (3)
C7B—H7BA	0.97	C7D—H7DA	0.97
C7B—H7BB	0.97	C7D—H7DB	0.97
C8B—C9B	1.525 (3)	C8D—C9D	1.525 (3)
C8B—H8BA	0.97	C8D—H8DA	0.97
C8B—H8BB	0.97	C8D—H8DB	0.97
C9B—C10B	1.527 (3)	C9D—C10D	1.523 (3)
C9B—H9BA	0.97	C9D—H9DA	0.97
C9B—H9BB	0.97	C9D—H9DB	0.97
C10B—H10D	0.96	C10D—H10J	0.96
C10B—H10E	0.96	C10D—H10K	0.96
C10B—H10F	0.96	C10D—H10L	0.96
			
C11A—O2A—H2A	109.5	C11C—O2C—H2C	109.5
O4A—N1A—O3A	122.31 (17)	O4C—N1C—O3C	122.07 (16)
O4A—N1A—C5A	119.18 (16)	O4C—N1C—C5C	119.29 (16)
O3A—N1A—C5A	118.50 (17)	O3C—N1C—C5C	118.63 (17)
C6A—N2A—C7A	124.42 (17)	C6C—N2C—C7C	122.69 (16)
C6A—N2A—H2AB	117.8	C6C—N2C—H2CB	118.7
C7A—N2A—H2AB	117.8	C7C—N2C—H2CB	118.7
C2A—C1A—C6A	121.92 (17)	C2C—C1C—C6C	122.32 (18)
C2A—C1A—H1AA	119.0	C2C—C1C—H1CA	118.8
C6A—C1A—H1AA	119.0	C6C—C1C—H1CA	118.8
C1A—C2A—C3A	120.92 (18)	C1C—C2C—C3C	120.92 (18)
C1A—C2A—H2AA	119.5	C1C—C2C—H2CA	119.5
C3A—C2A—H2AA	119.5	C3C—C2C—H2CA	119.5
C4A—C3A—C2A	118.93 (19)	C4C—C3C—C2C	118.71 (19)
C4A—C3A—C11A	120.07 (17)	C4C—C3C—C11C	121.61 (17)
C2A—C3A—C11A	121.00 (17)	C2C—C3C—C11C	119.68 (17)
C3A—C4A—C5A	120.41 (18)	C3C—C4C—C5C	120.46 (18)
C3A—C4A—H4AA	119.8	C3C—C4C—H4CA	119.8
C5A—C4A—H4AA	119.8	C5C—C4C—H4CA	119.8
C4A—C5A—C6A	122.44 (17)	C4C—C5C—C6C	122.16 (17)
C4A—C5A—N1A	116.05 (17)	C4C—C5C—N1C	116.21 (17)
C6A—C5A—N1A	121.51 (18)	C6C—C5C—N1C	121.63 (18)
N2A—C6A—C1A	120.29 (17)	N2C—C6C—C1C	119.90 (17)
N2A—C6A—C5A	124.49 (18)	N2C—C6C—C5C	124.72 (17)
C1A—C6A—C5A	115.21 (18)	C1C—C6C—C5C	115.37 (18)
N2A—C7A—C8A	110.12 (16)	N2C—C7C—C8C	111.33 (16)
N2A—C7A—H7AA	109.6	N2C—C7C—H7CA	109.4
C8A—C7A—H7AA	109.6	C8C—C7C—H7CA	109.4
N2A—C7A—H7AB	109.6	N2C—C7C—H7CB	109.4
C8A—C7A—H7AB	109.6	C8C—C7C—H7CB	109.4
H7AA—C7A—H7AB	108.1	H7CA—C7C—H7CB	108.0
C9A—C8A—C7A	112.02 (16)	C7C—C8C—C9C	110.83 (16)
C9A—C8A—H8AA	109.2	C7C—C8C—H8CA	109.5
C7A—C8A—H8AA	109.2	C9C—C8C—H8CA	109.5
C9A—C8A—H8AB	109.2	C7C—C8C—H8CB	109.5
C7A—C8A—H8AB	109.2	C9C—C8C—H8CB	109.5
H8AA—C8A—H8AB	107.9	H8CA—C8C—H8CB	108.1
C8A—C9A—C10A	112.47 (17)	C10C—C9C—C8C	113.28 (17)
C8A—C9A—H9AA	109.1	C10C—C9C—H9CA	108.9
C10A—C9A—H9AA	109.1	C8C—C9C—H9CA	108.9
C8A—C9A—H9AB	109.1	C10C—C9C—H9CB	108.9
C10A—C9A—H9AB	109.1	C8C—C9C—H9CB	108.9
H9AA—C9A—H9AB	107.8	H9CA—C9C—H9CB	107.7
C9A—C10A—H10A	109.5	C9C—C10C—H10G	109.5
C9A—C10A—H10B	109.5	C9C—C10C—H10H	109.5
H10A—C10A—H10B	109.5	H10G—C10C—H10H	109.5
C9A—C10A—H10C	109.5	C9C—C10C—H10I	109.5
H10A—C10A—H10C	109.5	H10G—C10C—H10I	109.5
H10B—C10A—H10C	109.5	H10H—C10C—H10I	109.5
O1A—C11A—O2A	123.22 (19)	O1C—C11C—O2C	122.87 (19)
O1A—C11A—C3A	120.06 (17)	O1C—C11C—C3C	121.59 (17)
O2A—C11A—C3A	116.72 (17)	O2C—C11C—C3C	115.54 (17)
C11B—O2B—H2B	109.5	C11D—O2D—H2D	109.5
O3B—N1B—O4B	121.60 (16)	O3D—N1D—O4D	121.99 (17)
O3B—N1B—C5B	118.91 (17)	O3D—N1D—C5D	118.31 (17)
O4B—N1B—C5B	119.48 (16)	O4D—N1D—C5D	119.70 (16)
C6B—N2B—C7B	123.53 (16)	C6D—N2D—C7D	123.37 (16)
C6B—N2B—H2BB	118.2	C6D—N2D—H2DB	118.3
C7B—N2B—H2BB	118.2	C7D—N2D—H2DB	118.3
C2B—C1B—C6B	122.43 (18)	C2D—C1D—C6D	122.42 (17)
C2B—C1B—H1BA	118.8	C2D—C1D—H1DA	118.8
C6B—C1B—H1BA	118.8	C6D—C1D—H1DA	118.8
C1B—C2B—C3B	120.95 (18)	C1D—C2D—C3D	120.55 (18)
C1B—C2B—H2BA	119.5	C1D—C2D—H2DA	119.7
C3B—C2B—H2BA	119.5	C3D—C2D—H2DA	119.7
C4B—C3B—C2B	118.65 (18)	C4D—C3D—C2D	118.67 (18)
C4B—C3B—C11B	120.77 (17)	C4D—C3D—C11D	121.47 (17)
C2B—C3B—C11B	120.58 (17)	C2D—C3D—C11D	119.86 (17)
C5B—C4B—C3B	120.75 (17)	C3D—C4D—C5D	121.11 (18)
C5B—C4B—H4BA	119.6	C3D—C4D—H4DA	119.4
C3B—C4B—H4BA	119.6	C5D—C4D—H4DA	119.4
C4B—C5B—C6B	121.82 (17)	C4D—C5D—C6D	121.58 (17)
C4B—C5B—N1B	116.48 (16)	C4D—C5D—N1D	117.00 (17)
C6B—C5B—N1B	121.71 (17)	C6D—C5D—N1D	121.42 (17)
N2B—C6B—C1B	120.51 (17)	N2D—C6D—C1D	120.10 (17)
N2B—C6B—C5B	124.17 (17)	N2D—C6D—C5D	124.32 (17)
C1B—C6B—C5B	115.32 (18)	C1D—C6D—C5D	115.58 (17)
N2B—C7B—C8B	110.78 (16)	N2D—C7D—C8D	110.88 (16)
N2B—C7B—H7BA	109.5	N2D—C7D—H7DA	109.5
C8B—C7B—H7BA	109.5	C8D—C7D—H7DA	109.5
N2B—C7B—H7BB	109.5	N2D—C7D—H7DB	109.5
C8B—C7B—H7BB	109.5	C8D—C7D—H7DB	109.5
H7BA—C7B—H7BB	108.1	H7DA—C7D—H7DB	108.1
C7B—C8B—C9B	111.18 (16)	C7D—C8D—C9D	111.18 (16)
C7B—C8B—H8BA	109.4	C7D—C8D—H8DA	109.4
C9B—C8B—H8BA	109.4	C9D—C8D—H8DA	109.4
C7B—C8B—H8BB	109.4	C7D—C8D—H8DB	109.4
C9B—C8B—H8BB	109.4	C9D—C8D—H8DB	109.4
H8BA—C8B—H8BB	108.0	H8DA—C8D—H8DB	108.0
C8B—C9B—C10B	112.58 (17)	C10D—C9D—C8D	112.85 (17)
C8B—C9B—H9BA	109.1	C10D—C9D—H9DA	109.0
C10B—C9B—H9BA	109.1	C8D—C9D—H9DA	109.0
C8B—C9B—H9BB	109.1	C10D—C9D—H9DB	109.0
C10B—C9B—H9BB	109.1	C8D—C9D—H9DB	109.0
H9BA—C9B—H9BB	107.8	H9DA—C9D—H9DB	107.8
C9B—C10B—H10D	109.5	C9D—C10D—H10J	109.5
C9B—C10B—H10E	109.5	C9D—C10D—H10K	109.5
H10D—C10B—H10E	109.5	H10J—C10D—H10K	109.5
C9B—C10B—H10F	109.5	C9D—C10D—H10L	109.5
H10D—C10B—H10F	109.5	H10J—C10D—H10L	109.5
H10E—C10B—H10F	109.5	H10K—C10D—H10L	109.5
O1B—C11B—O2B	123.35 (19)	O1D—C11D—O2D	122.66 (18)
O1B—C11B—C3B	120.57 (18)	O1D—C11D—C3D	121.40 (17)
O2B—C11B—C3B	116.07 (17)	O2D—C11D—C3D	115.94 (17)
			
C6A—C1A—C2A—C3A	1.9 (3)	C6C—C1C—C2C—C3C	0.4 (3)
C1A—C2A—C3A—C4A	1.6 (3)	C1C—C2C—C3C—C4C	1.2 (3)
C1A—C2A—C3A—C11A	−177.65 (17)	C1C—C2C—C3C—C11C	−177.89 (17)
C2A—C3A—C4A—C5A	−2.3 (3)	C2C—C3C—C4C—C5C	−0.8 (3)
C11A—C3A—C4A—C5A	176.92 (17)	C11C—C3C—C4C—C5C	178.28 (17)
C3A—C4A—C5A—C6A	−0.4 (3)	C3C—C4C—C5C—C6C	−1.2 (3)
C3A—C4A—C5A—N1A	179.24 (16)	C3C—C4C—C5C—N1C	178.17 (16)
O4A—N1A—C5A—C4A	167.58 (17)	O4C—N1C—C5C—C4C	175.51 (16)
O3A—N1A—C5A—C4A	−13.0 (2)	O3C—N1C—C5C—C4C	−5.2 (2)
O4A—N1A—C5A—C6A	−12.8 (3)	O4C—N1C—C5C—C6C	−5.1 (3)
O3A—N1A—C5A—C6A	166.64 (17)	O3C—N1C—C5C—C6C	174.24 (16)
C7A—N2A—C6A—C1A	5.5 (3)	C7C—N2C—C6C—C1C	−0.2 (3)
C7A—N2A—C6A—C5A	−173.76 (17)	C7C—N2C—C6C—C5C	−179.46 (17)
C2A—C1A—C6A—N2A	176.23 (18)	C2C—C1C—C6C—N2C	178.34 (18)
C2A—C1A—C6A—C5A	−4.4 (3)	C2C—C1C—C6C—C5C	−2.3 (3)
C4A—C5A—C6A—N2A	−176.99 (18)	C4C—C5C—C6C—N2C	−177.97 (17)
N1A—C5A—C6A—N2A	3.4 (3)	N1C—C5C—C6C—N2C	2.7 (3)
C4A—C5A—C6A—C1A	3.7 (3)	C4C—C5C—C6C—C1C	2.7 (3)
N1A—C5A—C6A—C1A	−175.93 (16)	N1C—C5C—C6C—C1C	−176.62 (16)
C6A—N2A—C7A—C8A	−179.73 (17)	C6C—N2C—C7C—C8C	−174.03 (17)
N2A—C7A—C8A—C9A	−178.77 (17)	N2C—C7C—C8C—C9C	176.71 (16)
C7A—C8A—C9A—C10A	−178.04 (18)	C7C—C8C—C9C—C10C	179.19 (18)
C4A—C3A—C11A—O1A	−176.17 (18)	C4C—C3C—C11C—O1C	−177.83 (19)
C2A—C3A—C11A—O1A	3.1 (3)	C2C—C3C—C11C—O1C	1.3 (3)
C4A—C3A—C11A—O2A	3.8 (3)	C4C—C3C—C11C—O2C	1.9 (3)
C2A—C3A—C11A—O2A	−176.93 (17)	C2C—C3C—C11C—O2C	−179.03 (17)
C6B—C1B—C2B—C3B	−0.4 (3)	C6D—C1D—C2D—C3D	−0.2 (3)
C1B—C2B—C3B—C4B	−2.0 (3)	C1D—C2D—C3D—C4D	−1.9 (3)
C1B—C2B—C3B—C11B	177.20 (17)	C1D—C2D—C3D—C11D	178.42 (17)
C2B—C3B—C4B—C5B	2.1 (3)	C2D—C3D—C4D—C5D	1.6 (3)
C11B—C3B—C4B—C5B	−177.16 (17)	C11D—C3D—C4D—C5D	−178.79 (17)
C3B—C4B—C5B—C6B	0.3 (3)	C3D—C4D—C5D—C6D	0.9 (3)
C3B—C4B—C5B—N1B	−179.51 (16)	C3D—C4D—C5D—N1D	−178.39 (16)
O3B—N1B—C5B—C4B	10.2 (3)	O3D—N1D—C5D—C4D	8.8 (3)
O4B—N1B—C5B—C4B	−170.94 (17)	O4D—N1D—C5D—C4D	−171.89 (17)
O3B—N1B—C5B—C6B	−169.60 (16)	O3D—N1D—C5D—C6D	−170.48 (16)
O4B—N1B—C5B—C6B	9.3 (3)	O4D—N1D—C5D—C6D	8.9 (3)
C7B—N2B—C6B—C1B	−3.1 (3)	C7D—N2D—C6D—C1D	−3.3 (3)
C7B—N2B—C6B—C5B	176.30 (17)	C7D—N2D—C6D—C5D	176.76 (17)
C2B—C1B—C6B—N2B	−177.90 (18)	C2D—C1D—C6D—N2D	−177.50 (17)
C2B—C1B—C6B—C5B	2.6 (3)	C2D—C1D—C6D—C5D	2.4 (3)
C4B—C5B—C6B—N2B	177.98 (17)	C4D—C5D—C6D—N2D	177.14 (18)
N1B—C5B—C6B—N2B	−2.3 (3)	N1D—C5D—C6D—N2D	−3.6 (3)
C4B—C5B—C6B—C1B	−2.5 (3)	C4D—C5D—C6D—C1D	−2.8 (3)
N1B—C5B—C6B—C1B	177.21 (16)	N1D—C5D—C6D—C1D	176.42 (16)
C6B—N2B—C7B—C8B	176.51 (17)	C6D—N2D—C7D—C8D	177.42 (17)
N2B—C7B—C8B—C9B	−178.18 (17)	N2D—C7D—C8D—C9D	−177.64 (16)
C7B—C8B—C9B—C10B	−178.45 (18)	C7D—C8D—C9D—C10D	177.78 (18)
C4B—C3B—C11B—O1B	176.75 (18)	C4D—C3D—C11D—O1D	177.59 (18)
C2B—C3B—C11B—O1B	−2.5 (3)	C2D—C3D—C11D—O1D	−2.8 (3)
C4B—C3B—C11B—O2B	−3.0 (3)	C4D—C3D—C11D—O2D	−2.4 (3)
C2B—C3B—C11B—O2B	177.81 (17)	C2D—C3D—C11D—O2D	177.29 (17)

### Hydrogen-bond geometry (Å, °)

**Table:**

*D*—H···*A*	*D*—H	H···*A*	*D*···*A*	*D*—H···*A*
O2A—H2A···O1A^i^	0.82	1.80	2.613 (2)	168
O2B—H2B···O1D^ii^	0.82	1.81	2.624 (2)	172
O2C—H2C···O1C^iii^	0.82	1.80	2.619 (2)	175
O2D—H2D···O1B^iv^	0.82	1.80	2.612 (2)	173
C1A—H1AA···O3A^v^	0.93	2.46	3.290 (2)	149
C1C—H1CA···O3C^vi^	0.93	2.44	3.256 (2)	146
C1D—H1DA···O3B^vii^	0.93	2.40	3.227 (2)	148
N2A—H2AB···O4A	0.86	2.02	2.656 (2)	130
N2B—H2BB···O4B	0.86	2.01	2.651 (2)	130
N2C—H2CB···O4C	0.86	2.02	2.649 (2)	129
N2D—H2DB···O4D	0.86	2.01	2.649 (2)	130
C1B—H1BA···O3D	0.93	2.49	3.311 (2)	147
C7D—H7DA···Cg1^v^	0.97	2.81	3.584 (2)	137
C7C—H7CB···Cg2^viii^	0.97	2.88	3.621 (2)	134

Symmetry codes: (i) −*x*+1, −*y*, −*z*+1; (ii) *x*, −*y*+1/2, *z*−1/2; (iii) −*x*, −*y*, −*z*+1; (iv) *x*, −*y*+1/2, *z*+1/2; (v) −*x*+1, *y*+1/2, −*z*+1/2; (vi) −*x*, *y*+1/2, −*z*+1/2; (vii) *x*, *y*+1, *z*; (viii) −*x*, *y*−1/2, −*z*+1/2.
